# How does the external context affect an implementation processes? A qualitative study investigating the impact of macro-level variables on the implementation of goal-oriented primary care

**DOI:** 10.1186/s13012-024-01360-0

**Published:** 2024-04-16

**Authors:** Ine Huybrechts, Anja Declercq, Emily Verté, Peter Raeymaeckers, Sibyl Anthierens, on behalf of the Primary Care Academy, Roy Remmen, Emily Verté, Muhammed Mustafa Sirimsi, Peter Van Bogaert, Hans De Loof, Kris Van den Broeck, Sibyl Anthierens, Ine Huybrechts, Peter Raeymaeckers, Veerle Bufel, Dirk Devroey, Bert Aertgeerts, Birgitte Schoenmakers, Lotte Timmermans, Veerle Foulon, Anja Declerq, Dominique Van de Velde, Pauline Boeckxstaens, An De Sutter, Patricia De Vriendt, Lies Lahousse, Peter Pype, Dagje Boeykens, Ann Van Hecke, Peter Decat, Rudi Roose, Sandra Martin, Erica Rutten, Sam Pless, Anouk Tuinstra, Vanessa Gauwe, Leen Van Landschoot, Maja Lopez Hartmann, Tony Claeys, Hilde Vandenhoudt, Kristel De Vliegher, Susanne Op de Beeck

**Affiliations:** 1 Department of Family Medicine and Population Health, University of Antwerp, Doornstraat 331, 2610 Antwerp, Belgium; 2 Department of Family Medicine and Chronic Care, Vrije Universiteit Brussel, Laarbeeklaan 103, 1090 Jette/Brussels, Belgium; 3 LUCAS — Centre for Care Research and Consultancy, KU Leuven, Minderbroedersstraat 8/5310, 3000 Leuven, Belgium; 4 Center for Sociological Research, Faculty of Social Sciences, KU Leuven, Parkstraat 45/3601, 3000 Leuven, Belgium; 5 Department of Social Work, University of Antwerp, St-Jacobstraat 2, 2000 Antwerp, Belgium

**Keywords:** Contingency theory, External context, Goal-oriented care, Institutional theory, Primary care, Implementation process, Macro-context, Network theory, Organizational theories, Resource dependency theory

## Abstract

**Background:**

Although the importance of context in implementation science is not disputed, knowledge about the actual impact of external context variables on implementation processes remains rather fragmented. Current frameworks, models, and studies merely describe macro-level barriers and facilitators, without acknowledging their dynamic character and how they impact and steer implementation. Including organizational theories in implementation frameworks could be a way of tackling this problem. In this study, we therefore investigate how organizational theories can contribute to our understanding of the ways in which external context variables shape implementation processes. We use the implementation process of goal-oriented primary care in Belgium as a case.

**Methods:**

A qualitative study using in-depth semi-structured interviews was conducted with actors from a variety of primary care organizations. Data was collected and analyzed with an iterative approach. We assessed the potential of four organizational theories to enrich our understanding of the impact of external context variables on implementation processes. The organizational theories assessed are as follows: institutional theory, resource dependency theory, network theory, and contingency theory. Data analysis was based on a combination of inductive and deductive thematic analysis techniques using NVivo 12.

**Results:**

Institutional theory helps to understand mechanisms that steer and facilitate the implementation of goal-oriented care through regulatory and policy measures. For example, the Flemish government issued policy for facilitating more integrated, person-centered care by means of newly created institutions, incentives, expectations, and other regulatory factors. The three other organizational theories describe both counteracting or reinforcing mechanisms. The financial system hampers interprofessional collaboration, which is key for GOC. Networks between primary care providers and health and/or social care organizations on the one hand facilitate GOC, while on the other hand, technology to support interprofessional collaboration is lacking. Contingent variables such as the aging population and increasing workload and complexity within primary care create circumstances in which GOC is presented as a possible answer.

**Conclusions:**

Insights and propositions that derive from organizational theories can be utilized to expand our knowledge on how external context variables affect implementation processes. These insights can be combined with or integrated into existing implementation frameworks and models to increase their explanatory power.

Contributions to literature

- Knowledge on how external context variables affect implementation processes tends to be rather fragmented. Insights on external context in implementation research often remain limited to merely describing macro-context barriers and facilitators.
- Organizational theories contribute to our understanding on the impact of external context to an implementation process by explaining the complex interactions between organizations and their environments.
- Findings can be utilized to help explain the mechanism of change in an implementation process and can be combined with or integrated into existing implementation frameworks and models to gain a broader picture on how external context affects implementation processes.

## Background

In this study, we integrate organizational theories to provide a profound analysis on how external context influences the implementation of complex interventions. There is a growing recognition that the context in which an intervention takes place highly influences implementation outcomes [1, 2]. Despite its importance, researchers are challenged by the lack of a clear definition of context. Most implementation frameworks and models do not define context as such, but describe categories or elements of context, without capturing it as a whole [2, 3]. Studies often distinguish between internal and external context: micro- and meso-level internal context variables are specific to a person, team, or organization. Macro-level external context variables consist of variables on a broader, socio-economic and policy level that are beyond one’s control [4].

Overall, literature provides a rather fragmented and limited perspective on how external context influences the implementation process of a complex intervention. Attempts are made to define, categorize, and conceptualize external context [5, 6]. Certain implementation frameworks and models specifically mention external context, such as the conceptual model of evidence-based practice implementation in public service sectors [7], the Consolidated Framework for Implementation Research [8], or the i-PARiHS framework [9]. However, they remain limited to identifying and describing external context variables. Few studies are conducted that specifically point towards the actual impact of macro-level barriers and facilitators [10–12] but only provide limited insights in how these shape an implementation process. Nonetheless, external contextual variables can be highly disruptive for an organization’s implementation efforts, for example, when fluctuations in funding occur or when new legislation or technology is introduced [13]. In order to build a more comprehensive view on external context influences, we need an elaborative theoretical perspective.

### Organizational theories as a frame of reference

To better understand how the external context affects the implementation process of a primary care intervention, we build upon research of Birken et al. [13] who demonstrate the explanatory power of organizational theories. Organizational theories can help explain the complex interactions between organizations and their environments [13], providing understanding on the impact of external context on the mechanism of change in an implementation process. We focus on three of the theories Birken et al. [8] put forward: institutional theory, resource dependency theory, and contingency theory. We also include network theory in recognition of the importance of interorganizational context and social ties between various actors, especially in primary care settings which are characterized by a multitude of diverse actors (meaning: participants of a process).

These four organizational theories demonstrate the ways in which organizations interact with their external environment in order to sustain and fulfill their core activities. All four of them do this with a different lens. Institutional theory states that an organization will aim to fulfil the expectations, values, or norms that are posed upon them in order to achieve a fit with their environment [14]. This theory helps to understand the relationships between organizations and actors and the institutional context in which they operate. Institutions can broadly be defined as a set of expectations for social or organizational behavior that can take the form formal structures such as regulatory entities, legislation, or procedures [15]. Resource dependency theory explains actions and decisions of organizations in terms of their dependence on critical and important resources. It postulates that organizations will respond to their external environment to secure the resources they need to operate [16, 17]. This theory helps to gain insight in how fiscal variables can shape the adoption of an innovation. Contingency theory presupposes that an organizations’ effectiveness depends on the congruence between situational factors and organizational characteristics [18]. External context variables such as social and economic change and pressure can impact the way in which an innovation will be integrated. Lastly, network theory in its broader sense underlines the strength of networks: collaborating in networks can establish an effectiveness in which outcomes are achieved that could not be realized by individual organizations acting independently. Networks are about connecting or sharing information, resources, activities, and competences of three or more organizations aiming to achieve a shared goal or outcome [19, 20]. Investigating networks helps to gain understanding of the importance of the interorganizational context and how social ties between organizations affect the implementation process of a complex intervention.

### Goal-oriented care in Flanders as a case

In this study, we focus on the implementation of the approach goal-oriented care (GOC) in primary care in Flanders, the Dutch-speaking region in Belgium. Primary care is a highly institutionalized and regulated setting with a high level of professionalism. Healthcare organizations can be viewed as complex adaptive systems that are increasingly interdependent [21]. The primary care landscape in Flanders is characterized by many primary care providers (PCPs) being either self-employed or working in group practices or community health centers. They are organized and financed at different levels (federal, regional, local). In 2015–2019, a primary care reform was initiated in Flanders in which the region was geographically divided into 60 primary care zones that are governed by care councils. The Flemish Institute of Primary Care was created as a supporting institution aiming to strengthen the collaboration between primary care health and welfare actors. The complex and multisectoral nature of primary care in Flanders forms an interesting setting to gain understanding in how macro-level context variables affect implementation processes.

The concept of GOC implies a paradigm shift [22] that shifts away from a disease or problem-oriented focus towards a person-centered focus that departs from “what matters to the patient.” Boeykens et al. [23] state in their concept analysis that GOC could be described as a healthcare approach encompassing a multifaceted, dynamic, and iterative process underpinned by the patient’s context and values. The process is characterized by three stages: goal elicitations, goal setting, and goal evaluation in which patients’ needs and preferences form the common thread. It is an approach in which PCPs and patients collaborate to identify personal life goals and to align care with those goals [23]. An illustration of how this manifests at individual level can be found in Table 1. The concept of GOC was incorporated in Flemish policies and included in the primary care reform in 2015–2019. It has gained interest in research and policy as a potential catalyst for integrated care [24]. As such, the implementation of GOC in Flanders provides an opportunity to investigate the external context of a complex primary care intervention. Our main research question is as follows: what can organizational theories tell us about the influence of external context variables on the implementation process of GOC?

**Table 1:** Model case of Joseph to demonstrate GOC at the individual level

Joseph, 68 years old, suffers from diabetes, hypertension, and chronic obstructive pulmonary disease. Throughout his entire working life, he was a secondary school teacher. He has been retired for 3 years now *(patients’ context)*. Despite the fact that he is limited by his health condition, he loves spending time gardening and playing with his grandchildren *(patients’ needs and preferences)*. A few years ago, he was a passionate cyclist, but his racing bike has been stored for a long time now. His friends encourage him to cycle with them on a weekly basis *(patients’ context)*. His wife supports this initiative and argues that this will be beneficial for his social contact *(patients’ context)*. Every month, Joseph visits his family doctor for a check-up. For each consultation, he prepares a list of things he wants to discuss. He has the chance to share his story in an open communication in which trust and mutual respect are key components *(goal elicitation)*. In his monthly check-up with his family doctor, he suggests his wishes to cycle again with his friends (patients’ needs and preferences & goal setting; interaction). His doctor doubts whether this will be possible, and after discussion and negotiation *(goal setting; interaction)*, they plan that he would join his friends in their weekly cycling trip but only for the first 2 h *(goal setting; foundation for SMART goal)*. The group will be asked to adapt their pace, and Joseph will make sure that he does not need to return back home on his own. The doctor makes adjustments to the medication scheme according to the increased efforts Joseph will make *(goal setting; care plan)*. He will also contact the cardiologist to inform him about the changes to the medication schema *(goal setting; care plan)*. The family doctor and the cardiologist will collaborate in order to succeed in Joseph’s goal *(goal setting; care delivery)*. The family doctor and Joseph agree to discuss and evaluate the course after 3 months (goal evaluation; feedback & evaluation). It is possible to increase or decrease the intensity depending on Joseph’s health state and his own preferences *(goal evaluation; evaluation)*.	

Model case borrowed from Boeykens, D., Boeckxstaens, P., De Sutter, A., Lahousse, L., Pype, P., De Vriendt, P., & Van de Velde, D. (2022). Goal-oriented care for patients with chronic conditions or multimorbidity in primary care: a scoping review and concept analysis. PLoS One, 17(2), e0262843

## Methods

We assess the potential of four organizational theories to enrich our understanding of the impact of external context variables on implementation processes. The organizational theories assessed are as follows: institutional theory, resource dependency theory, network theory, and contingency theory. Qualitative research methods are most suitable to investigate such complex matters, as they can help answer “how” and “why” questions on implementation [25]. We conducted online, semi-structured in-depth interviews with various primary care actors. These actors all had some level of experience at either meso- or micro-level with GOC implementation efforts.

### Sample selection

For our purposive sample, we used the following inclusion criteria: 1) working in a Flemish health/social care context in which initiatives are taken to implement GOC and 2) having at least 6 months of experience. For recruitment, we made an overview of all possible stakeholders that are active in GOC by calling upon the network of the Primary Care Academy (PCA)^1^. Additionally, a snowballing approach was used in which respondents could refer to other relevant stakeholders at the end of each interview. This leads to respondents with different backgrounds (not only medical) and varying roles, such as being a staff member, project coordinator, or policy maker. We aimed at a maximum variation in the type of organizations which were represented by respondents, such as different governmental institutions and a variety of healthcare/social care organizations. In some cases, paired interviews were conducted [26] if the respondents were considered complementary in terms of expertise, background, and experience with the topic. An information letter and a request to participate was send to each stakeholder by e-mail. One reminder was sent in case of nonresponse.

### Data collection

Interviews were conducted between January and June 2022 by a sociologist trained in qualitative research methods. Interviewing took place online using the software Microsoft Teams and were audio-recorded and transcribed verbatim. A semi-structured interview guide was used, which included (1) an exploration of the concept of GOC and how the respondent relates to this topic, (2) questions on how GOC became a topic of interest and initiatives within the respondent’s setting, and (3) the perceived barriers and facilitators for implementation. An iterative approach was used between data collection and data analysis, meaning that the interview guide underwent minor adjustments based on proceeding insights from earlier interviews in order to get richer data.

### Data analysis

All data were thematically analyzed, both inductively and deductively, supported by the software NVivo 12©. For the inductive part, implicit and explicit ideas within the qualitative data were identified and described [27]. The broader research team, with backgrounds in sociology, medical sciences, and social work, discussed these initial analyses and results. The main researcher then further elaborated this into a broad understanding. This was followed by a deductive part, in which characteristics and perspectives from organizational theories were used as sensitizing concepts, inspired by research from Birken et al. [13]. This provided a frame of reference and direction, adding interpretive value to our analysis [28]. These analyses were subject of peer debriefing with our cooperating research team to validate whether these results aligned with their knowledge of GOC processes. This enhances the trustworthiness and credibility of our results [29, 30]. Data analysis was done in Dutch, but illustrative quotes were translated into English.

## Results

In-depth interviews were performed with *n* = 23 respondents (see Table 2): five interviews were duo interviews, and one interview took place with *n* = 3 respondents representing one organization. We had *n* = 6 refusals: *n* = 3 because of time restraints, *n* = 1 did not feel sufficiently knowledgeable about the topic, *n* = 1 changed professional function, and there was *n* = 1 nonresponse. Respondents had various ways in which they related towards the macro-context: we included actors that formed part of external context (e.g., the Flemish Agency of Care and Health), actors that facilitate and strengthen organizations in the implementation of GOC (e.g., the umbrella organization for community health centers), and actors that actively convey GOC inside and outside their setting (e.g., an autonomous and integral home care service). Interviews lasted between 47 and 72 min. Table 3 gives an overview on the main findings of our deductive analysis with their respective links to the propositions of each of the organizational theories that we applied as a lens.

**Table 2:** Overview of respondents and type of organization

Respondent ID	Type of organization	Professional function	Role of respondents with regard to GOC
INT1	Governmental institution	Staff member	Expanding awareness/knowledge
INT2	Governmental institution	Staff member	Expanding awareness/knowledge
INT3	Governmental institution	Policy officer	Creating preconditions
INT4	Governmental institution	Policy officer	Creating preconditions
INT5	Governmental institution	Policy officer	Creating preconditions
INT6	Nonprofit organization	Consultant	Creating preconditions
INT7	Umbrella organization	Staff member	Expanding awareness/knowledge
INT8	Umbrella organization	Staff member	Expanding awareness/knowledge
INT9	Provider organization	Project manager	Expanding awareness/knowledge
INT10	Provider organization	Project manager	Expanding awareness/knowledge
INT11	Provider organization	Chair	Expanding awareness/knowledge
INT12	Patient organization	Policy officer	Developing/offering tools
INT13	Patient organization	Director	Developing/offering tools
INT14	Health/social care organization	Staff member	Developing/offering tools
INT15	Health/social care organization	Director	Developing/offering tools
INT16	Health/social care organization	Coordinator	Coordinating
INT17	Health/social care organization	Counsellor	Coordinating
INT18	Nonprofit organization	Project coordinator	Expanding awareness/knowledge
INT19	Nonprofit organization	Project coordinator	Expanding awareness/knowledge
INT20	Primary care zone/care council	Coordinator	Networking
INT21	Primary care zone/care council	Staff member	Networking
INT22	Primary care zone/care council	Staff member	Networking
INT23	Primary care zone/care council	Coordinator	Networking

**Table 3:** Overview of organizational theory and applications to the implementation of GOC

Perspective	Propositions	Application to the implementation of GOC	Illustrative quotes
Institutional theory	Change is prompted by norms, values, and expectations deriving from the institutional environment	Belgian policy steers primary care actors towards person-centered, GOC by creating structures and actors that support its uptake and regulations that tie in with GOC	*This was a conscious effort to introduce it in primary care. An entire transition trajectory has been started since 2017. Integrated care, GOC are essential parts of that vision note. All our policies that we are rolling out are grafted on this. — INT04*
Resources dependency theory	Decisions and actions of organizations will be affected by the external resources they need to secure their activities	The current financial system (fee for service) does not support interprofessional collaboration nor GOC	*We will remain stuck as long as finances are based on the consult model and on performances.. this doesn’t promote GOC. — INT21*
Network theory	Actors that are connecting and working together to achieve a shared goal will attain outcomes more efficiently and integrated than actors working independently	Interprofessional collaboration and a shared language between different disciplines are a prerequisite for providing GOC. The newly established care councils are facilitators as they connect different PCPs and hereby put forward GOC as a common goal. However, the current available technology does not allow PCPs to collaborate according to a GOC with other disciplines	*The importance of this overarching part… I as a GP want to engage in GOC; you as a physiotherapist; a social worker wants to do it; a family care department wants to do it. But how do we do it together? — INT23*
Contingency theory	An organization adapts and responds to external context variables. The social and economic landscape can shape organizational decisions and actions	Social and demographic variables such as the aging population and the increasing pressure in terms of workload and complexity of patient profiles on healthcare can form the circumstances in which GOC can be a possible answer towards the emerging needs in the primary care context	*This partly has to do with our ageing population. We run into a shortage of capacity to provide care for them. And this will be the main driver towards change. — INT04*

### Institutional theory: laying foundations for a shift towards GOC

For the implementation of GOC in primary care, looking at the data with an institutional theory lens helps us understand the way in which primary care organizations will respond to social structures surrounding them. Institutional theory describes the influence of institutions, which give shape to organizational fields: “organizations *that, in the aggregate, constitute a recognized area of institutional life* [31], p. 148. Prevailing institutions within primary care in Flanders can affect how organizations within such organizational fields fulfil their activities. Throughout our interviews, we recognized several dynamics that are being described in institutional theory.

First of all, the changing landscape of primary care in Flanders (see 1.2) was often brought up as a dynamic in which GOC is intertwined with other changes. Respondents mention an overall tendency to reform primary care to becoming more integrated and the ideas of person-centered care becoming more upfront. These expectations in how primary care should be approached seem to affect the organizational field of primary care: “You could tell that in people’s minds they are ready to look into what it actually means to put the patient, the person central. — INT01” Various policy actors are committed to further steer towards these approaches: “the government has called it the direction that we all have to move towards. — INT23” It was part of the foundations for the most recent primary care reform, leading to the creation of demographic primary care zones governed by care councils and the Flemish Institute of Primary Care as supporting institution.

These newly established actors were viewed by our respondents as catalysts of GOC. They pushed towards the aims to depart from local settings and to establish connections between local actors. Overall, respondents emphasized their added value as they are close to the field and they truly connect primary care actors. “They [care councils] have picked up these concepts and have started working on it. At the moment they are truly the incubators and ecosystems, as they would call it in management slang. — INT04” For an innovation such as GOC to be diffused, they are viewed as the ideal actors who can function as a facilitator or conduit. They are uniquely positioned as they are closely in contact with the practice field and can be a top-down conduit for governmental actors but also are able to address the needs from bottom-up. “In this respect, people look at the primary care zones as the ideal partners. […] We can start bringing people together and have that helicopter view: what is it that truly connects you? — INT23” However, some respondents also mentioned their difficult governance structure due to representation of many disciplines and organizations.

Other regulatory factors were mentioned by respondents were other innovations or changes in primary care that were intentionally linked to GOC: e.g., the BelRAI^2^ or Flemish Social Protection^3^. “The government also provides incentives. For example, family care services will gradually be obliged to work with the BelRAI screener. This way, you actually force them to start taking up GOC. — INT23” For GOC to be embedded in primary care, links with other regulatory requirements can steer PCPs towards GOC. Furthermore, it was sometimes mentioned that an important step would be for the policy level to acknowledge GOC as quality of care and to include the concept in quality standards. This would further formalize and enforce the institutional expectation to go towards person-centered care.

Currently, a challenge on institutional level as viewed by most respondents is that GOC is not or only to a limited extent incorporated in the basic education of most primary care disciplines. This leads to most of PCPs only having a limited understanding of GOC and different disciplines not having a shared language in this matter. “You have these primary health and welfare actors who each have their own approach, history and culture. To bring them together and to align them is challenging. — INT10” The absence of GOC as a topic in basic education is mentioned by various respondents as a current shortcoming in effectively implementing GOC in the wider primary care landscape.

Overall, GOC is viewed as our respondents as a topic that has recently gained a lot interest, both by individual PCPS, organizations, and governmental actors. The Flemish government has laid some foundations to facilitate this change with newly created institutions and incentives. However, other external context variables can interfere in how the concept of GOC is currently being picked up and what challenges arise.

### Resource dependency theory: in search for a financial system that accommodates interprofessional collaboration

Another external context variable that affects how GOC can be introduced is the financial system that is at place. To analyze themes that were raised during the interviews with regard to finances, we utilized a resource dependency perspective. This theory presumes that organizations are dependent on financial resources and are seeking ways to ensure their continued functioning [16, 17]. To a certain extent, this collides with the assumptions of institutional theory that foregrounds organization’s conformity to institutional pressures [32]. Resource dependency theory in contrast highlights differentiation of organizations that seek out competitive advantages [32].

In this context, respondents mention that their interest and willingness to move towards a GOC approach are held back by the current dominant system of pay for performance in the healthcare system. This financial system is experienced as restrictive, as it does not provide any incentive to PCPs for interprofessional collaboration, which is key for GOC. A switch to a flat fee system (in which a fixed fee is charged for each patient) or bundled payment was often mentioned as desirable. PCPs and health/social care organizations working in a context where they are financially rewarded for a trajectory or treatment of a patient in its entirety ensure that there is no tension with their necessity to obtain financial resources, as described in the resource dependency theory. Many of our respondents voice that community health centers are a good example. They cover different healthcare disciplines and operate with a fixed price per enrolled patient, regardless of the number of services for that patient. This promotes setting up preventive and health-promoting actions, which confirms our finding on the relevance of dedicated funding.

At the governmental level, the best way to finance and give incentives is said to be a point of discussion: “For years, we have been arguing about how to finance. Are we going to fund counsel coordination? Or counsel organization? Or care coordination? — INT04” Macro-level respondents do however mention financial incentives that are already in place to stimulate interprofessional collaboration: fees for multidisciplinary consultation being the most prominent. Other examples were given in which certain requirements were set for funding (e.g., Impulseo^4^, VIPA^5^) that stimulate actors or settings in taking steps towards more interprofessional collaboration.

Nowadays, financial incentives to support organizations to engage in GOC tend to be project grants. However, a structural way to finance GOC approaches is currently lacking, according to our respondents. As a consequence, a long-term perspective for organizations is lacking; there is no stable financing and organizations are obliged to focus on projects instead of normalizing GOC in routine practice. According to a resource dependency perspective, the absence of financial incentives for practicing GOC hinders organizations in engaging with the approach, as they are focused on seeking out resources in order to fulfil their core activities.

### A network-theory perspective: the importance of connectedness for the diffusion of an innovation

Throughout the interviews, interorganizational contextual elements were often addressed. A network theory lens states that collaborating in networks can lead to outcomes that could not be realized by individual organizations acting independently [19, 20]. Networks consist of a set of actors such as PCPs or health/social care organizations along with a set of ties that link them [33]. These ties can be state-type ties (e.g., role based, cognitive) or event-type ties (e.g., through interactions, transactions). Both type of ties can enable a flow in which information or innovations can pass, as actors interact [33]. To analyze the implementation process of GOC and how this is diffused through various actors, a network theory perspective can help understand the importance of the connection between actors.

A first observation throughout the interviews in which we notice the importance of networks was in the mentioning of local initiatives that already existed before the creation of the primary care zones/care councils. In the area around Ghent, local multidisciplinary networks already organized community meetings, bringing together different PCPs on overarching topics relating to long-term care for patients with chronic conditions. These regions have a tradition of collaboration and connectedness of PCPs, which respondents mention to be highly valuable: “This ensures that we are more decisive, speaking from one voice with regards to what we want to stand for. — INT23” Respondents voice that the existence of such local networks has had a positive effect on the diffusion of ideas such as GOC, as trust between different actors was already established.

Further mentioning of the importance of networks could be found in respondents acknowledging one of the presumptions of network theory: working collaboratively towards a specific objective leads to outcomes that cannot be realized independently. This is especially true for GOC, an approach that in essence requires different disciplines to work together: “When only one GP, nurse or social worker starts working on it, it makes no sense. Everyone who is involved with that person needs to be on board. Actually, you need to finetune teams surrounding a person — INT11.” This is why several policy-level respondents mentioned that emphasis was placed on organizing GOC initiatives in a neighborhood-oriented way, in which accessible, inclusive care is aimed at by strengthening social cohesion. This way, different types of PCPs got to know each other through these sessions an GOC and would start to get aligned on what it means to provide GOC. However, in particular, self-employed PCPs are hard to reach. According to our respondents, occupational groups and care councils are suitable actors to engage these self-employed PCPs, but they are not always much involved in such a network*.*

To better connect PCPs and health/social care organizations, the absence of connectedness through the technological landscape is also mentioned. Current technological systems and platforms for documenting patient information do not allow for aligning and sharing between disciplines. In Flanders, there is a history of each discipline developing its own software, which lacks centralization or unification: “For years, they have decided to just leave it to the market, in such a way that you ended up with a proliferation of software, each discipline having its own package. — INT06” Most of the respondents mentioning this were aware that Flanders government is currently working on a unified digital care and support platform and were optimistic about its development.

### Contingency theory: how environmental pressure can be a trigger for change

Our interviews were conducted during a rather dynamic and unique period of time in which the impact of social change and pressure was clearly visible: the Flemish primary care reform was ongoing which leads to the creation of care councils and VIVEL (see 3.1.1), and the COVID crisis impacted the functioning of these and other primary care actors. These observed effects of societal changes are reminiscent of the assumptions that are made in contingency theory. In essence, contingency theory presupposes that “organizational effectiveness results from fitting characteristics of the organization, such as its structure, to contingencies that reflect the situation of the organization [34], p. 1.” When it comes to the effects of the primary care reform and the COVID crisis, there were several mentions on how primary care actors reorganized their activities to adapt to these circumstances. Representatives of care councils/primary care zones whom we interviewed underlined that they were just at the point where they could again engage with their original action plans, not having to take up so many COVID-related tasks anymore. On the one hand, the COVID crisis had however forced them to immediately become functional and has also contributed that various primary care actors quickly got to know them. On the other hand, the COVID crisis has also kept them from their core activities for a while. On top of that, the crisis has also triggered a change the overall view towards data sharing. Some respondents mention a rather protectionist approach towards data sharing, while data sharing has become more normalized during the COVID crisis. This discussion was also relevant for the creation of a unified shared patient record in terms of documenting and sharing patient goals.

Other societal factors that were mentioned having an impact on the uptake of GOC are the demographic composition of a certain area. It was suggested that areas that are characterized by a patient population with more chronic care needs will be more likely to steer towards GOC as a way of coping with these complex cases. “You always have these GPs who blow it away immediately and question whether this is truly necessary. They will only become receptive to this when they experience needs for which GOC can be a solution — INT11.” On a macro-level, several respondents have mentioned how a driver for change is to have the necessity for change becoming very tangible. As PCPs are confronted with increasing numbers of patients with complex, chronic needs and their work becomes more demanding, the need for change becomes more acute. This finding is in line with what contingency theory underlines: changes in contingency (e.g., the population that is increasingly characterized by aging and multimorbidity) are an impetus for change for health/social care organizations to resolve this by adopting a structure that better fits the current environmental characteristics [34].

## Discussion

**Fig. 1:**
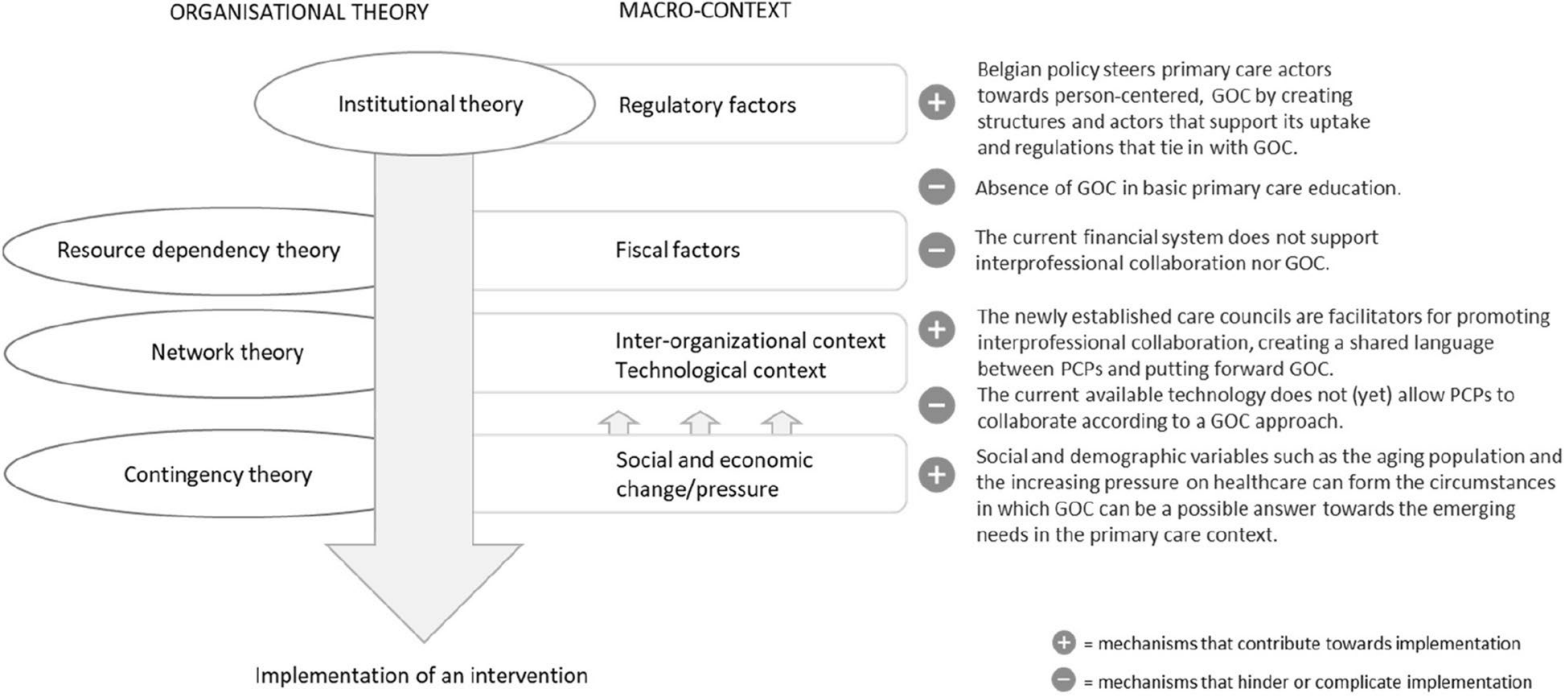
How organizational theories can help explain the way in which macro-level context variables affect implementation of an intervention

Our research demonstrates the applicability of organizational theories to help explain the impact that macro-level context variables have on an implementation process. These insights can be integrated into existing implementation frameworks and models to add the explanatory power of macro-level context variables, which is to date often neglected. The organizational theories demonstrate the ways in which organizations interact with their external environment in order to sustain and fulfill their core activities. As demonstrated in Fig. 1, institutional theory largely explains how social expectations in the form of institutions lead towards the adoption or implementation of innovation, such as GOC. However, other organizational theories demonstrate how other macro-context elements on different areas can either strengthen or hamper the implementation process.

Departing from the mechanisms that are postulated by institutional theory, we observed that the shift towards GOC is part of a larger Flemish primary care reform in which and new institutions have been established and polices have been drawn up to go towards more integrated, person-centered care. To achieve this, governmental actors have placed emphasis on socialization of care, the local context, and establishing ties between organizations in order to become more complementary in providing primary health care [35]. With various initiatives surrounding this aim, the Flemish government is steering towards GOC. This is reminiscent of the mechanisms that are posed within institutional theory: organizations adapt to prevailing norms and expectations and mimic behaviors that are surrounding them [15, 36].

Throughout our data, we came across concrete examples of how institutionalization takes place. DiMaggio and Powell [31] describe the subsequent process of isomorphism: organizations start to resemble each other as they are conforming to their institutional environment. A first mechanism through which this change occurs is coercive isomorphism and is clearly noticeable in our data. This type of isomorphism results from both formal and informal pressure coming from organizations from which a dependency relationship exists and from cultural expectations in the society [31]. Person-centered, GOC care is both formally propagated by governmental institutions and procedures and informally expected by current social tendencies. Care councils within primary care zones explicitly propagate and disseminate ideas and approaches that are desirable on policy level. Another form of isomorphism is professional isomorphism and relates to our finding that incorporation of GOC in basic education is currently lacking. The presumptions of professional isomorphism back up the importance of this: values, norms, and ideas that are developed during education are bound to find entrance within organizations as professionals start operating along these views.

Although many observations in our data back up the assumptions of institutional theory, it should be noticed that new initiatives such as the promotion of person-centered care and GOC can collide with earlier policy trends. Martens et al. [12] have examined the Belgian policy process relating three integrated care projects and concluded that although there is a strong support for a change towards a more patient-centered system, the current provider-driven system and institutional design complicate this objective. Furthermore, institutional theory tends to simplify actors as passive adopters of institutional norms and expectations and overlook the human agency and sensemaking that come with it [37]. For GOC, it is particularly true that PCPs will actively have to seek out their own style and fit the approach in their own way of working. Moreover, GOC was not just addressed as a governmental expectation but for many PCPs something they inherently stood behind.

Resources dependency theory poses that organizations are dependent on critical resources and adapt their way of working in response to those resources [17]. From our findings, it seems that the current financial system does not promote GOC, meaning that the mechanisms that are put forward in resources dependency theory are not set in motion. A macro-level analysis of barriers and facilitators in the implementation of integrated care in Belgium by Danhieux et al. [10] also points towards the financial system and data sharing as two of the main contextual determinants that affect implementation.

Throughout our data, the importance of a network approach was frequently mentioned. Interprofessional collaboration came forward as a prerequisite to make GOC happen, as well as active commitment on different levels. Burns, Nembhard, and Shortell [38] argue that research efforts on implementing person-centered, integrated care should have more focus on the use of social networks to study relational coordination. In terms of interprofessional collaboration, to date, Belgium has a limited tradition of working team-based with different disciplines [35]. However, when it comes to strengthening a cohesive primary care network, the recently established care councils have become an important facilitator. As a network governance structure, they resemble mostly a Network Administrative Organization (NAO): a separate, centralized administrative entity that is externally governed and not another member providing its own services [19]. According to Provan and Kenis [19], this type of governance form is most effective in a rather dense network with many participants, when the goal consensus is moderately high, characteristics that are indeed representative for the Flemish primary care landscape. This strengthens our observation that care councils have favorable characteristics and are well-positioned to facilitate the interorganizational context to implement GOC.

Lastly, the presumptions within contingency theory became apparent as respondents talked about how the need for change needs to become tangible for PCPs and organizations to take action, as they are increasingly faced with a shortage of time and means and more complex patient profiles. Furthermore, De Maeseneer [39] affirms our findings that the COVID-19 crisis could be employed as an opportunity to strengthen primary health care, as health becomes prioritized and its functioning becomes re-evaluated. Overall, contingency theory can help gain insight in how and why certain policy trends or decisions are made. A study of Bruns et al. [40] found that modifiable external context variables such as interagency collaboration were predictive for policy support for intervention adoption, while unmodifiable external context variable such as socio-economic composition of a region was more predictive for fiscal investments that are made.

### Strengths and limitations

This study contributes to our overall understanding of implementation processes by looking into real-life implementation efforts for GOC in Flanders. It goes beyond a mere description of external context variables that affect implementation processes but aims to grasp which and how external context variables influence implementation processes. A variety of respondents from different organizations, with different backgrounds and perspectives, were interviewed, and results were analyzed by researchers with backgrounds in sociology, social work, and medical sciences. Results can not only be applied to further develop sustainable implementation plans for GOC but also enhance our understanding of how the external context influences and shapes implementation processes. As most research on contextual variables in implementation processes has until now mainly focused on internal context variables, knowledge on external context variables contributes to gaining a bigger picture of the mechanism of change.

However, this study is limited to the Flemish landscape, and external context variables and their dynamics might differ from other regions or countries. Furthermore, our study has examined and described how macro-level context variables affect the overall implementation processes of GOC. Further research is needed on the link between outer and inner contexts during implementation and sustainment, as explored by Lengninck-Hall et al. [41]. Another important consideration is that our sample only includes the “believers” in GOC and those who are already taking steps towards its implementation. It is possible that PCPs themselves or other relevant actors who are more skeptical about GOC have a different view on the policy and organizational processes that we explored. Furthermore, data triangulations in which this data is complemented with document analysis could have expanded our understanding and verified subjective perceptions of respondents.

## Conclusion

Insights and propositions that derive from organizational theories can be utilized to expand our knowledge on how external context variables affect implementation processes. Our research demonstrates that the implementation of GOC in Flanders is steered and facilitated by regulatory and policy variables, which sets in motion mechanisms that are described in institutional theory. However, other external context variables interact with the implementation process and can further facilitate or hinder the overall implementation process. Assumptions and mechanisms explained within resource dependency theory, network theory, and contingency theory contribute to our understanding on how fiscal, technological, socio-economic, and interorganizational context variables affect an implementation process.

## Data Availability

The datasets generated and/or analyzed during the current study are not publicly available due to confidentiality guaranteed to participants but are available from the corresponding author on reasonable request.

## Abbreviations

GOC: Goal-oriented care
PCP: Primary care provider
PCA: Primary Care Academy

## Footnotes

1 The Primary Care Academy (PCA) is a research and teaching network of four Flemish universities, six university colleges, the White and Yellow Cross (an organization for home nursing), and patient representatives that have included GOC as one of their main research domains.

2 BelRAI, the Belgian implementation of the interRAI assessment tools; these are scientific, internationally validated instruments enabling an assessment of social, psychological, and physical needs and possibilities of individuals in different care settings. The data follows the person and is shared between care professionals and care organizations.

3 The Flemish Social Protection is a mandatory insurance established by the Flemish government to provide a range of concessions to individuals with long-term care and support needs due to illness or disability.

4 Impulseo, financial support for general practitioners who start an individual practice or join a group practice

5 VIPA, grants for the realization of sustainable, accessible, and affordable healthcare infrastructure
